# The effect of insulin on equine lamellar basal epithelial cells mediated by the insulin-like growth factor-1 receptor

**DOI:** 10.7717/peerj.5945

**Published:** 2018-11-29

**Authors:** Courtnay L. Baskerville, Subu Chockalingham, Patricia A. Harris, Simon R. Bailey

**Affiliations:** 1 Faculty of Veterinary and Agricultural Sciences, University of Melbourne, Melbourne, VIC, Australia; 2 Equine Studies Group, WALTHAM Centre for Pet Nutrition, Melton Mowbray, Leicestershire, UK

**Keywords:** Laminitis, Equine, IGF-1, Epithelium

## Abstract

**Background:**

In horses and ponies, insulin dysregulation leading to hyperinsulinemia may be associated with increased risk of laminitis, and prolonged infusion of insulin can induce the condition. It is unclear whether insulin may have a direct or indirect effect on the lamellar tissues. Insulin is structurally related to insulin-like growth factor (IGF-1), and can bind the IGF-1 receptor, albeit at a lower affinity than IGF-1.

**Methods:**

Immunohistochemistry was performed on formalin-fixed lamellar tissue sections from six normal horses, euthanised for non-research purposes, using an anti-IGF-1 receptor antibody. In further studies, lamellar epithelial cells were obtained by collagenase digestion from the hooves of 18 normal horses, also euthanised for non-research purposes, and incubated for 48 h in the presence of insulin (0–2,000 m IU/ml). The increase in cell numbers was determined using a cell proliferation assay, and compared to the effect of zero insulin using one-way ANOVA.

**Results:**

Immunohistochemistry demonstrated IGF-1 receptors on lamellar epidermal epithelial cells. With cultured cells, insulin caused a concentration-dependent increase in cell proliferation compared to untreated cells (maximal effect 63.3 ± 12.8% more cells after 48 h with 1,000 m IU/ml insulin; *P* < 0.01). Co-incubation with a blocking antibody against the IGF-1 receptor significantly inhibited the proliferative effect of insulin (*P* < 0.01).

**Discussion:**

These results demonstrate that IGF-1 receptors are present on lamellar epithelial cells. At high physiological concentrations, insulin may activate these cells, by a mechanism involving IGF-1 receptors, resulting in a proliferative effect. This mechanism could help to explain the link between hyperinsulinemia and laminitis.

## Introduction

It has become clear in recent years that the form of laminitis seen commonly in ponies and horses associated with repeated, prolonged or severe hyperinsulinaemia, so-called ‘endocrinopathic laminitis’ (McGowan, 2010), may be distinct in several respects to the inflammatory form of the disease which occurs secondary to carbohydrate overload in the hind gut or in the black walnut model (Katz & Bailey, 2012; Patterson-Kane, Karikoski & McGowan, 2018). Insulin dysregulation is thought to be the key factor linking the metabolic phenotype of obesity and insulin resistance with the increased risk of laminitis (comprising the features of Equine Metabolic Syndrome; EMS) (Frank et al., 2010). Laminitis associated with EMS is thought to be the most common form of laminitis and may be the underlying mechanism in pasture laminitis, although this has yet to be proven (Geor, 2010). It has been shown that laminitis can be induced in healthy ponies after prolonged administration of insulin (Asplin et al., 2007), and the apparently direct causal link between insulin and laminitis was subsequently confirmed in (non-insulin resistant) Standardbred horses (De Laat et al., 2010).

The histopathology observed in the feet of these insulin-infused horses showed particular characteristics which were distinct from the mainly inflammatory changes and widespread epidermal separation from the basement membrane seen in other forms of laminitis. Secondary epidermal lamellae (SEL) were significantly elongated (Karikoski et al., 2014) and lesions included swelling and disorganisation of epithelial cells plus increased mitotic activity (Asplin et al., 2010). A further ultrastructural study also found that prolonged hyperinsulinaemia was associated with unique characteristics including many basal epithelial cells being in mitosis and a decreased number of hemidesmosomes per unit length of basement membrane (Nourian et al., 2009). These changes were hypothesised to cause weakening of the lamellar attachments.

The mechanism(s) by which high concentrations of insulin might cause laminitis are still unclear. Previously it was thought that the associated insulin resistance might impair glucose delivery to the lamellar tissues, or affect blood flow (McGowan, 2010); however, it now appears to be a direct effect of insulin (although other factors may also play a role). The problem with this hypothesis though is that there appear to be no insulin receptors on the key cells which form the interface between the lamellar epidermis and dermis, namely the basal lamellar epithelial cells (Burns et al., 2013). Insulin receptors are only found on the vascular endothelium within the dermis.

However, insulin at high concentrations has commonly been found to have a proliferative effect on several different cell types and is often used in cell culture media for this purpose (Vigneri, Squatrito & Sciacca, 2010; Straus, 1984). It exerts this effect via its actions on the insulin-like growth factor-1 (IGF-1) receptor, since insulin and IGF-1 share considerable homology (Laron, 2004). These similarities mean that there is considerable overlap between the insulin and IGF-1 systems, and although the IGF-1 receptor has an affinity for insulin which is many fold lower than for IGF-1, in many species high concentrations of insulin will bind to and activate the IGF-1 receptor (Vigneri, Squatrito & Sciacca, 2010; De Meyts & Whittaker, 2002). In 2009 it was first proposed by Bailey & Chockalingham (2009) that insulin may be capable of stimulating a proliferative response in the lamellar epithelium via the IGF-1 receptor. Burns et al. (2013) subsequently found that the IGF-1 receptor was distributed on laminar epidermal epithelial cells as well as dermal vascular cells in ponies fed a high non-structural carbohydrate diet.

Furthermore, in horses which developed laminitis after receiving infusions of insulin, gene expression for the IGF-1 receptor in the lamellae was significantly decreased, thought to be associated with receptor down-regulation due to the high insulin levels and prolonged ligand binding of the receptors (De Laat et al., 2013).

The biological functions of IGF-1 receptor activation include stimulating cellular proliferation as well as cellular differentiation, migration and survival, all of which occur through ligand induced IGF-1 receptor (IGF-1R) activation (Duan, Ren & Gao, 2010). This may be consistent with many of the changes observed following insulin-induced laminitis. After the ligand binds to the IGF-1R, a signal transduction cascade is initiated through pathways including the extracellular signal-regulated kinase pathway (ERK 1/2; also known as p42/44 mitogen-activated protein kinase) (Rhodes, 2000; LeRoith & Roberts, 2003). It has previously been hypothesised that the increase in epidermal basal cell mitosis could be initiated through pathways such as the MAP-kinase pathway (Asplin et al., 2010).

The aim of the present study was firstly to confirm the presence of the IGF-1R in the lamellae of normal horses by immunohistochemistry (IHC) and then to use cultured lamellar epithelial cells in vitro to investigate the effect of increasing insulin concentrations on cellular proliferation and the importance of the ERK 1/2 pathway. Our hypothesis was that insulin selectively stimulates cellular proliferation of the lamellar basal epidermal epithelial cells, mediated via IGF-1 receptors and this is associated with the activation of the intracellular signalling pathway involving ERK1/2 (p42/44 MAP-kinase).

## Materials and Methods

Horse forelimbs were obtained from a local abattoir. All animals were apparently healthy and were euthanised for non-research purposes; the hooves were normal and showed no external gross evidence of laminitis. Distal limbs were removed immediately after euthanasia and placed on ice during transportation to the laboratory.

### Immunohistochemistry

Tissue sections of lamellae from beneath the dorsal hoof wall were obtained using a bandsaw, according to the methods of Pollitt (1996). One centimetre square sections were fixed in 10% formalin for 48 h and then transferred to 95% ethanol. After embedding in paraffin wax, five μm sections were cut and prepared on glass slides.

Sections were deparaffinised and rehydrated (Yoon, Berger & Roser, 2011), and then rinsed in distilled water and heated in antigen-retrieval solution (Dako, North Sydney, NSW, Australia) as described previously by Pearl et al. (2007). Sections were then incubated with 3% peroxidase solution and blocked with 4% bovine serum albumin (BSA) in phosphate buffered saline (PBS) for 30 min. Sections were then incubated in either chicken polyclonal anti-IGF-1R primary antibody (Abcam, Cambridge, UK) (diluted 1:50 in PBS containing 1% BSA) or isotype-matched control antibody (chicken IgY; Abcam, Cambridge, UK) and incubated in a moist environment overnight at 4 °C. Following incubation with the primary antibodies, sections were then incubated with a universal secondary antibody amplification solution (Vectastain Universal Quick Kit; Vector Labs, Peterborough, UK). During subsequent treatment with 3,3′-diaminobenzidine (DAB) substrate-chromogen solution (Sigma-Aldrich, Castle Hill, NSW, Australia), development was monitored under a light microscope. Samples were then counterstained, dehydrated, cleared in xylene and mounted on slides with dibutylphthalate polystyrene xylene (DPX) mounting medium (Leica Biosystems, Mount Waverley, VIC, Australia). Slides were assessed for IGF-1R distribution by light microscopy.

### Primary culture of equine laminar epithelial cells

After splitting the dorsal hoof wall with a bandsaw, lamellar tissue was harvested in a sterile manner by peeling off the hoof wall from the underlying dermis, according to the methods of Medina-Torres et al. (2011). After the laminar tissue underneath the hoof wall was exposed, epidermal lamellar tissue was scraped using a sterile scalpel blade and placed directly into sterile saline containing penicillin and streptomycin. The tissue was incubated in collagenase solution (Type II collagenase from Clostridium histolyticum; Sigma-Aldrich, Sydney, NSW, Australia; 50 mg in 50 mL PBS) for 3 h at 37 °C after which the cell suspension was filtered through a 70 μm cell strainer (Medina-Torres et al., 2011). Cells were then centrifuged at 300×*g* for 10 min at 20 °C, the supernatant was removed and cells were resuspended in sterile saline. After a further wash, the cells were resuspended in cell culture medium. Fibroblasts and other non-epithelial cells were removed by positive selection of epithelial cells on magnetic beads (Easysep biotin positive selection kit; StemCell Technologies, Vancouver, BC, Canada). Cells were incubated with the anti-IGF-1 receptor antibody and goat anti-chicken IgY biotinylated secondary antibody (Abcam, Cambridge, UK) before magnetic separation and re-suspension in culture medium.

Lamellar epithelial cells were then seeded into 75 cm flasks containing 20 mL of Dulbecco’s modified eagle medium (DMEM) (Gibco^®^ Life Technologies Australia Pty Ltd, Mulgrave, VIC, Australia) with 10% foetal calf serum, 1% Penicillin-Streptomycin and 0.25% Insulin 100 IU/mL and 0.4 μg/mL hydrocortisone, according to Visser & Pollitt (2010). Cells were maintained at 37 °C with 5% CO_2_ until approximately 80% confluent. At this point cells were passaged and resuspended in DMEM without insulin or hydrocortisone so that they became quiescent.

### Cellular proliferation assays

After cells were plated onto a 96 well plate and incubated overnight, the DMEM was again replaced with 100 μL of fresh DMEM, including 1% equine serum from a fasted Standardbred horse (serum insulin < 5 μIU/mL). Insulin was then added to duplicate wells of cells from *n* = 18 horses, at concentrations of 50, 100, 250, 500, 1,000 or 2,000 μIU/mL and incubated for 48 h at 37 °C with 5% CO_2_. Some wells of cells (from *n* = 12 horses) were also incubated with an anti-IGF-1 receptor antibody (chicken polyclonal; Abcam; 1:50 dilution) as a blocking antibody to prevent insulin binding to the IGF-1 receptor. An isotype control antibody (chicken IgY; Abcam, Cambridge, UK) was also used. Recombinant human IGF-1 (Abcam; one or five ng/mL concentration) was also added as a positive control (*n* = 6 horses).

Following incubation, 10 μL of MTT solution (TACS^™^ MTT Cellular Proliferation and Viability Assay; R&D Systems, Minneapolis, MN, USA) was added to each well and incubated for 4 h. The detergent reagent was then added and the cells were allowed to lyse overnight, producing a homogenous colour in each well. Each plate was then read with an automated microplate reader (Synergy H1 Hybrid Microplate Reader; Biotek, Winooski, VT, USA) with Gen5 Microplate Reader Software at 450 nm with a reference range of 650 nm.

### Western blotting for ERK 1/2 signalling

After a separate set of assays using the same conditions, cells (from six horses; *n* = 6) were washed with sterile phosphate buffered saline and then lysed with a proprietary lysis buffer (CelLytic; Sigma-Aldrich, Sydney, NSW, Australia). Protein concentration of each sample was determined using a BCA protein assay (Pierce BCA protein assay kit; ThermoFisher Scientific, Scoresby, VIC, Australia). Laemmli’s sample buffer was then added to give equivalent protein concentrations in each sample the tubes heated to 95 °C for 5 min and then 30 μg of protein was loaded onto a 10% acrylamide gel. The gel was run at 100 V and the proteins transferred onto a nitrocellulose membrane. The membrane was blocked in 10% milk in TBS-Tween for 1 h and then incubated with anti-phospho ERK 1/2 primary antibodies (rabbit polyclonal antibody; Abcam, Melbourne, VIC, Australia) at 1:500 dilution in 1% BSA for 2 h at room temperature. After washing, the membrane was placed in a secondary antibody (HRP-conjugated donkey anti-rabbit IgG; Abcam, Melbourne, VIC, Australia) at 1:1,000 dilution in 1% BSA for 2 h. The membrane finally developed using ECL (electrocheminulescent) solution (Pierce ECL Plus; ThermoFisher Scientific, Scoresby, VIC, Australia) and the film was then scanned and analysed by densitometric analysis (Unscanit Gel software ver 5.1; Silk Scientific Inc, Orem, UT, USA). Blots were stripped using stripping buffer (Chemicon ReBlot Plus; Merck Millipore, Bayswater, VIC, Australia) and then re-probed using a primary antibody against total ERK 1/2 (rabbit polyclonal antibody; Abcam, Melbourne, VIC, Australia).

### Statistical analysis

The normal distribution of the data collected from the cellular proliferation assays was verified by the Shapiro-Wilkes normality test. A one-way ANOVA with Dunnett’s multiple comparison test was performed to compare the proliferative effects of insulin at each concentration, compared to the control wells. Statistical comparisons were performed using Graphpad Prism version 5 (Graphpad Software Inc, La Jolla, CA, USA) and significant differences were accepted at *P* < 0.05.

## Results

### Immunohistochemistry for the IGF-1 receptor

**Figure 1:**
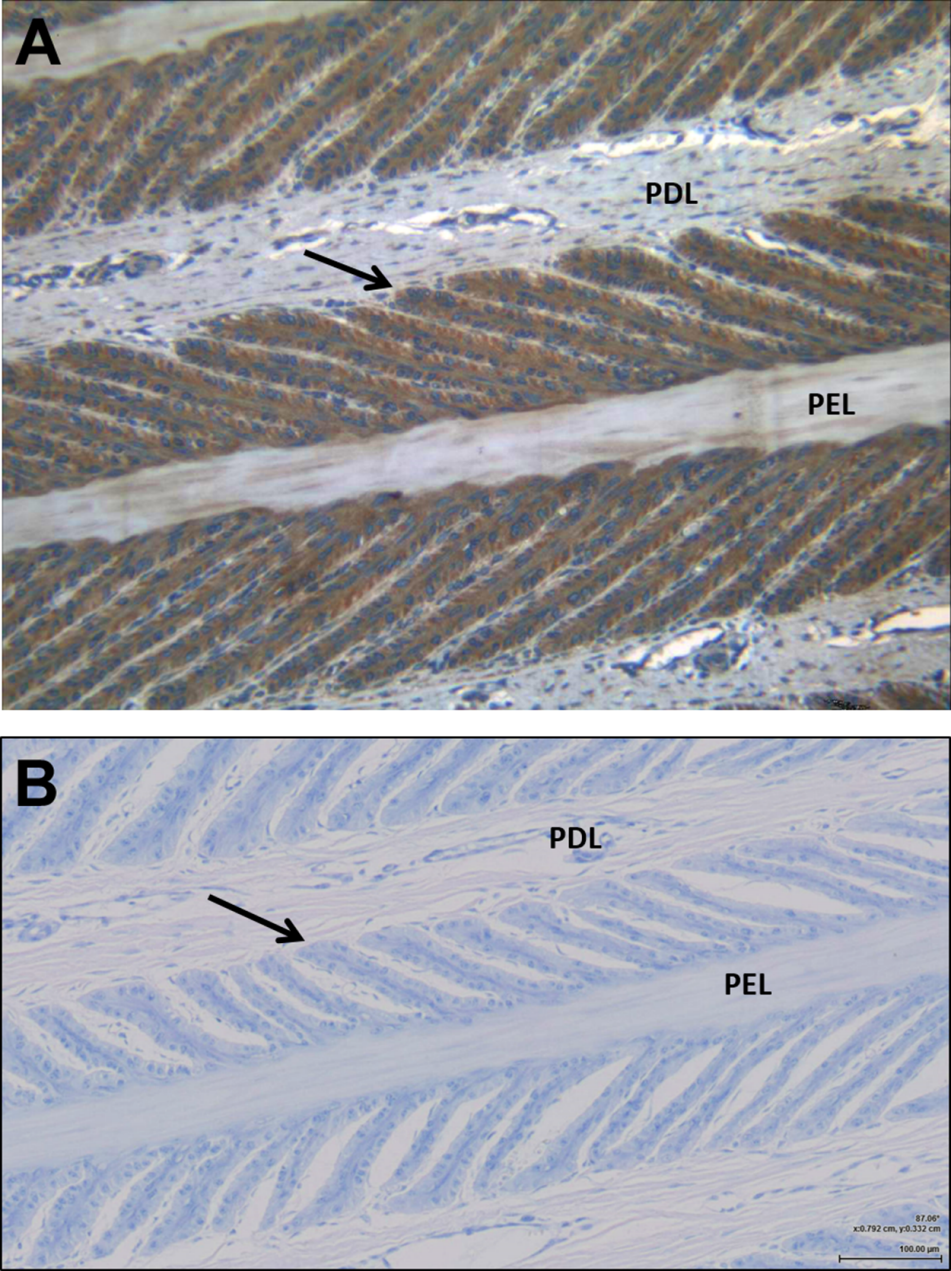
Immunohistochemistry showing the presence of IGF-1 receptors on the lamellar epithelial cells of a normal horse. (A) demonstrates the IGF-1R on the lamellar epithelial cells, compared to (B) the non-specific binding. Primary dermal lamellae (PDL) and primary epidermal lamellae (PEL) are indicated, and the arrows identify the basal epithelial cells of a secondary epidermal lamella.

Examination by light microscopy clearly demonstrated the presence of the IGF-1 receptor on the epithelial cells of the SEL (Fig. 1). Some faint staining of vascular endothelial and smooth muscle cells was noted, but otherwise there was little staining of cells within the dermal lamellar tissues. Control sections incubated in the absence of the primary antibody showed no non-specific staining by the secondary antibody.

### Cell proliferation

**Figure 2:**
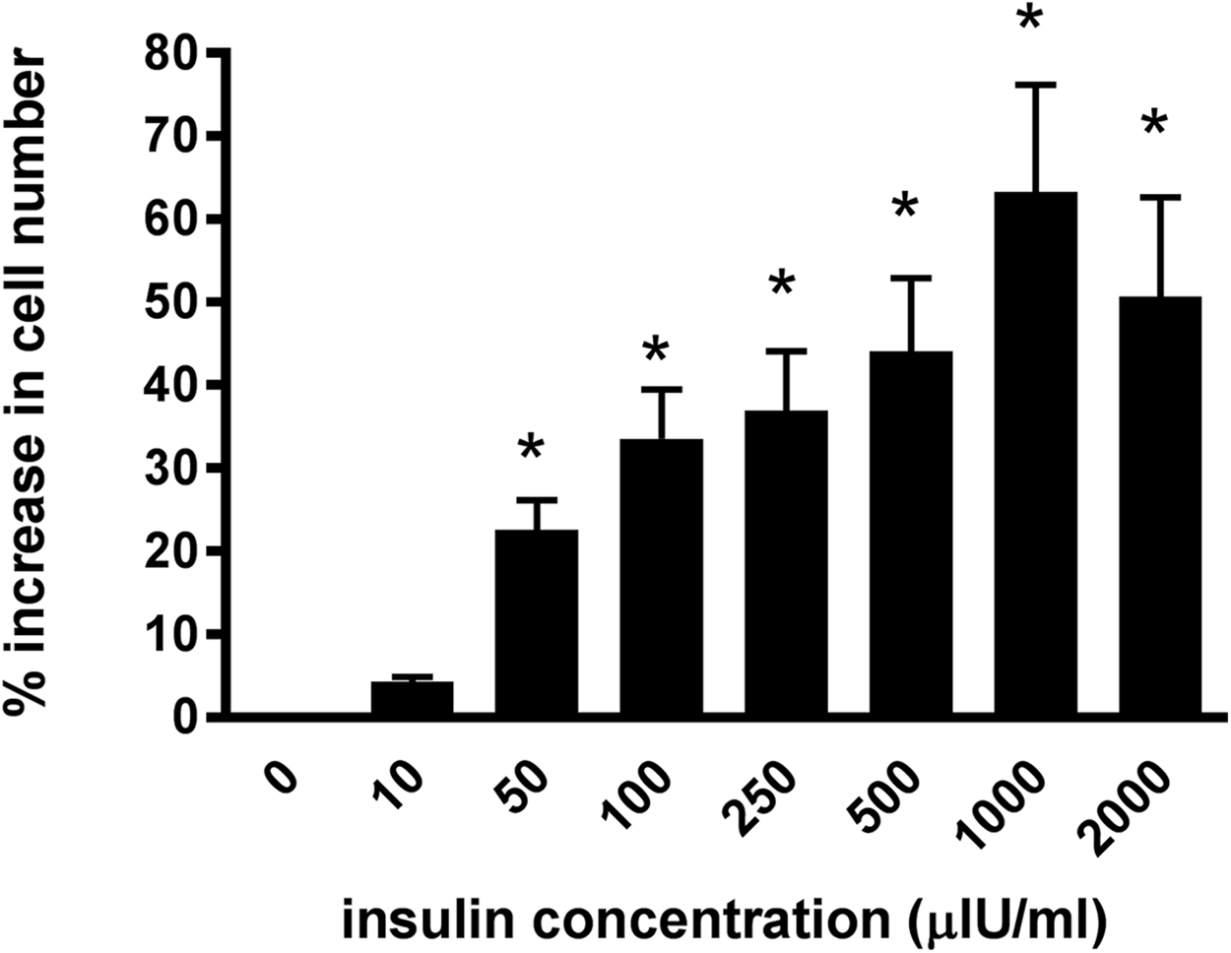
The effect of increasing insulin concentration on cellularproliferation of equine lamellar epithelial cells. Responses are expressed as the percentage increase in cellular proliferation compared with untreated control cells. Each bar represents mean ± SEM of percentage increase vs. zero insulin. * indicates statistically significant difference compared with control (*P* < 0.0001).

Incubating the lamellar epithelial cells in culture in the presence of insulin caused a concentration-dependent increase in cell number (Fig. 2). A significant effect of insulin was observed from 50 μIU/mL (*P* < 0.01) and the maximum effect (63.3 ± 12.8%) was evident at an insulin concentration of 1,000 μIU/mL.

**Figure 3:**
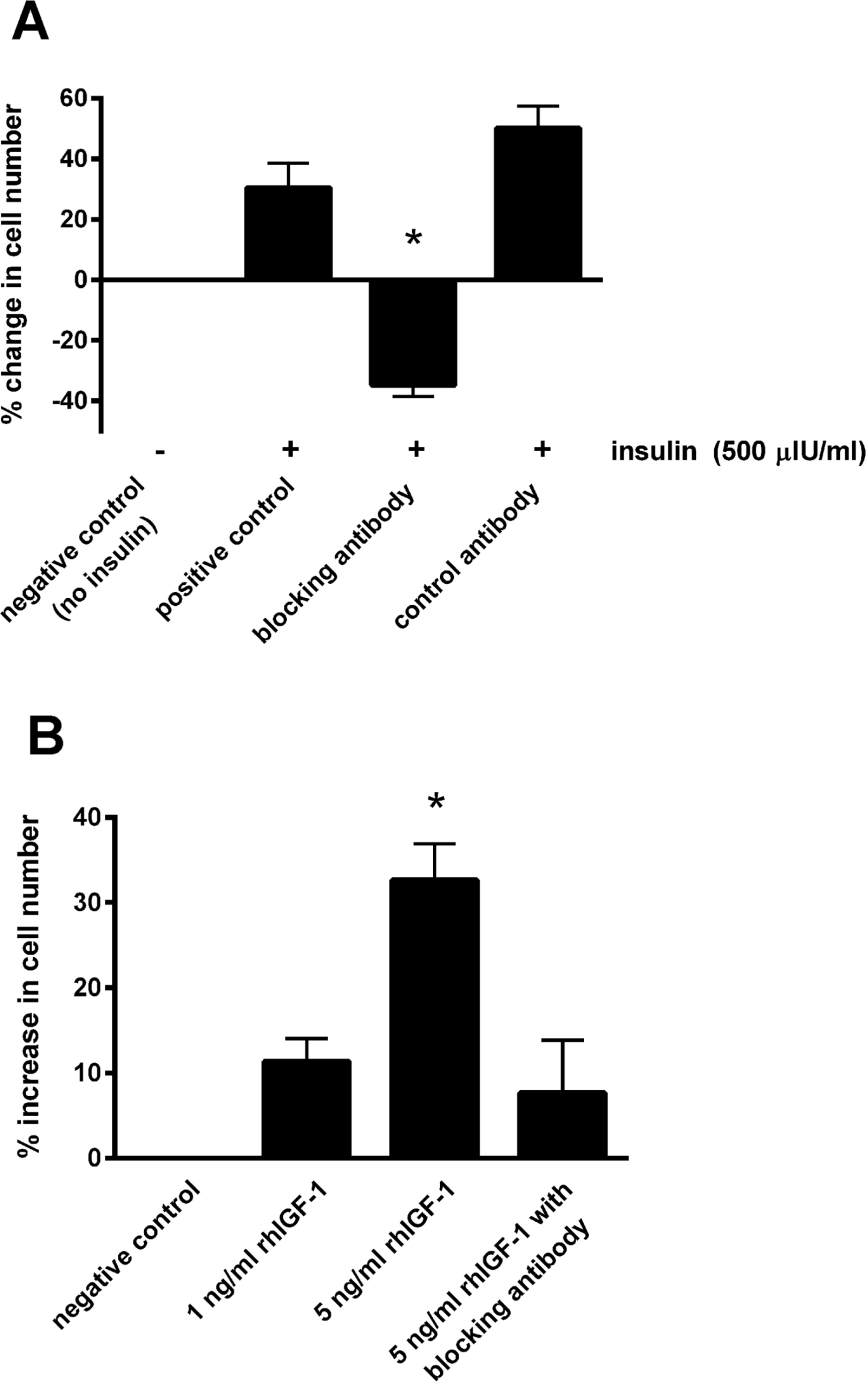
The effect of a blocking antibody on the proliferative response to insulin or IGF-1 in cultured equine lamellar epithelial cells. (A) Cells from 12 horses were incubated with insulin at either zero μIU/mL (negative control) or 500 μIU/mL (positive control), and further aliquots were incubated with 500 μIU/mL insulin in the presence of an anti-IGF-1 receptor blocking antibody or control antibody. (B) Cells from six horses were incubated with either zero, one or five ng/mL recombinant human IGF-1 and further aliquots were incubated with five ng/ml recombinant human IGF-1 in the presence of the anti-IGF-1 receptor blocking antibody. Each bar represents mean ± SEM of percentage change in cell number vs. zero insulin. * indicates statistically significant difference compared with control (*P*-value < 0.0001).

Blocking the IGF-1 receptors on the epithelial cells using the blocking antibody prevented the increase in cell number in the presence of 500 μIU/mL insulin (Fig. 3A). Incubating with the control antibody failed to elicit a significant effect. Furthermore, incubating cells with recombinant human IGF-1 caused a concentration-dependent proliferative effect, shown by a 32.7 ± 4.3% increase in cell number with the five ng/mL concentration (*P* < 0.05; Fig. 3B). Again the blocking antibody (anti-IGF-1 receptor) prevented the effect of this concentration of recombinant IGF-1.

### ERK activation

**Figure 4:**
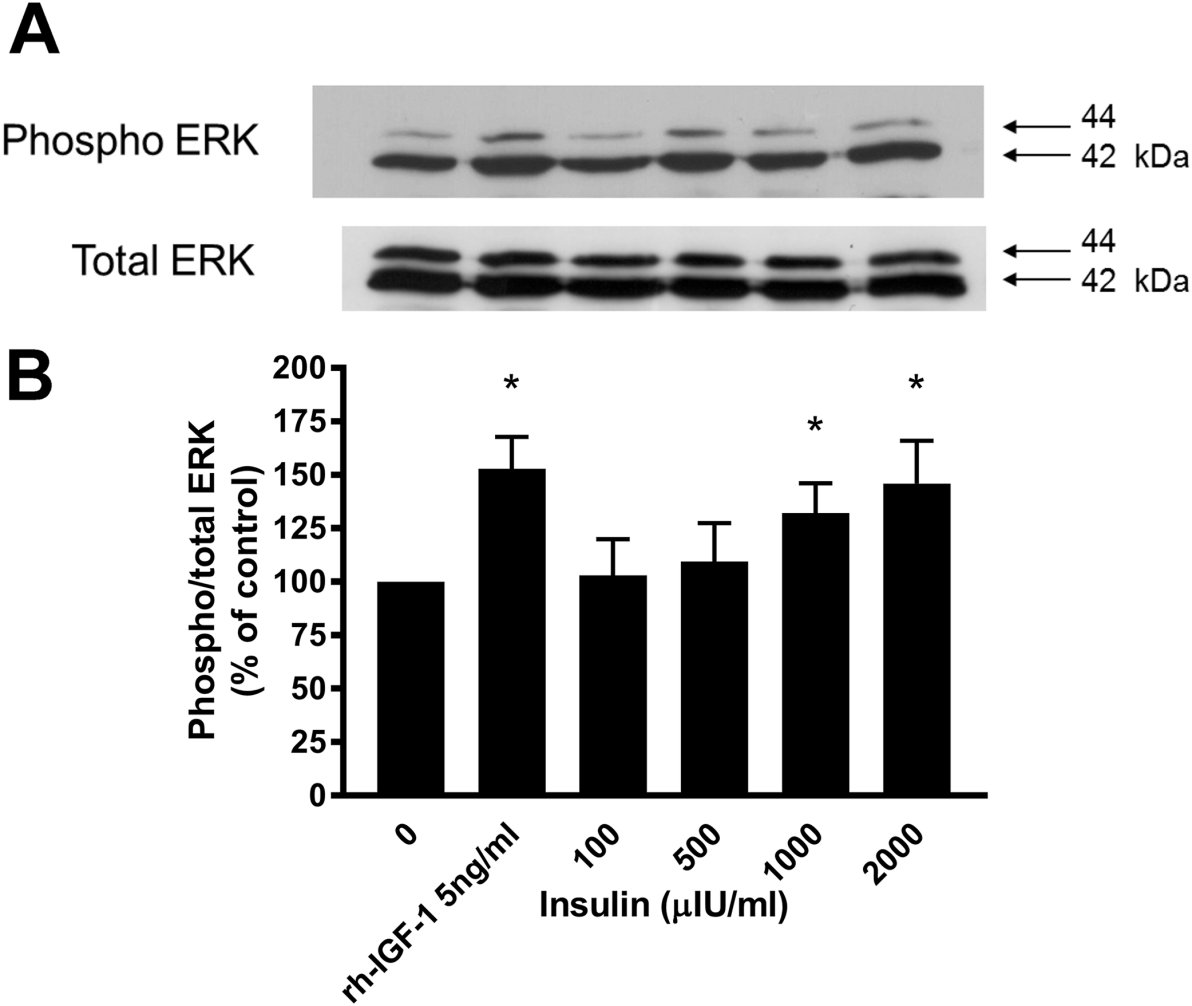
Effect of rhIGF-1 and insulin on ERK1/2 activation in equine lamellar basal epithelial cells. The cells were incubated with either rhIGF-1 or insulin, and ERK1/2 activation was then assessed by Western blotting. (A) Blots were probed for the phosphorylated (activated) form of the enzyme and expressed as a ratio of active: total enzyme. (B) The graph represents the ERK activity (mean ± SEM) as a percentage of the activity in the control (unstimulated) cells. * indicates statistically significant difference compared with control (*P* < 0.05).

Western blotting demonstrated that incubation of lamellar epithelial cells with insulin also caused activation of the ERK1/2 (P42/44 MAPK) cell signalling pathway (Fig. 4). Insulin concentrations of 1,000 and 2,000 μIU/mL resulted in a significantly increased ratio of phospho:total ERK (concentration-dependent increase), as did recombinant human IGF-1 (five ng/mL).

## Discussion

The findings of this study provide one possible explanation regarding the mechanism by which insulin may directly stimulate, or affect the function of, the lamellar basal epithelial cells, which is consistent with the observations from previous insulin-induced laminitis studies. In the in vivo studies, not only did high plasma concentrations of insulin induce laminitis consistently in both insulin-resistant and non-insulin resistant breeds (Asplin et al., 2007; De Laat et al., 2010), apparently in the absence of large changes in blood glucose, but the changes observed within the lamellar tissues specifically involved the basal epithelial cells and included increased mitotic figures (Asplin et al., 2010). Since the absence of insulin receptors on these key cells has been demonstrated by other groups (Burns et al., 2013) as well as our own laboratory (see Supplemental File), the most likely alternative receptor type for which insulin may act as a ligand is the IGF-1 receptor (Kullmann et al., 2015).

Therefore, the first step was to confirm the presence and distribution of the IGF-1 receptor on the lamellar epithelial cells of normal horses in situ. The IHC findings revealed that IGF-1 receptors are present in significant numbers on the basal epithelial cells of equine lamellae from normal (non-laminitic) horses. There was little staining of cells within the dermal lamellar tissues, although some positive staining was detected in vascular endothelium and vascular smooth muscle cells within the dermis. This confirms the findings of a previous study in ponies rather than horses, which found positive staining for IGF-1 receptor on epithelial cells, vascular cells and fibroblasts (Burns et al., 2013). A direct comparison between the chicken anti-IGF1R antibody used in the current study with the rabbit polyclonal antibody used previously in other studies (Burns et al., 2013; Yoon, Berger & Roser, 2011) is provided in the Supplemental File. Gene expression for the IGF-1 receptor has also been quantified in lamellar tissues from these animals (Kullmann et al., 2015).

Receptors for the peptide growth factor IGF-1 are found mainly in skeletal muscle, bone and cartilage, but also on many epithelial cells, and increased levels of this mediator have been associated with developmental osteochondral lesions in growing horses (Verwilghen et al., 2009). It has been suggested that IGF-1 plays a critical role in the expression and synthesis of collagen type II as well as protecting chondrocytes from apoptosis. The presence of IGF-1 receptors on the epithelial cells of the epidermal lamellae suggests that IGF-1 may be an important factor regulating either growth or lengthening of the hoof wall. It has been suggested that most hoof wall growth originates at the papillae of the coronary band (Daradka & Pollitt, 2004), although for the hoof wall to grow downwards, the cells of the SEL must detach from, and re-attach to, the basement membrane that connects the epidermis to the dermis (Mungall, Pollitt & Collins, 1998). This process might involve a response to IGF receptor stimulation, perhaps along with local collagenase enzyme activity (Mungall, Pollitt & Collins, 1998).

Although the affinity of insulin for the IGF-1 receptor is likely to be far less than the affinity of IGF-1 for its own receptor, there may be considerable crosstalk between the two systems, particularly when insulin concentrations are very high (Vigneri, Squatrito & Sciacca, 2010; Laron, 2004). Indeed, insulin is commonly added to cell culture media to promote cell growth and proliferation (Straus, 1984). In the present study, since cell proliferation is the most easily measured response to growth factor receptor activation, the effects of insulin were assessed on this response. Furthermore, the concentration range was chosen to be comparable to the plasma concentrations achieved in the infusion experiments that caused laminitis (Asplin et al., 2007; De Laat et al., 2010), and to cover the plasma concentrations that might be achieved post-prandially in ponies with insulin dysregulation that may be at risk of laminitis (Borer et al., 2012).

Incubating the cultured lamellar epithelial cells in the presence of insulin caused a concentration-dependent proliferative effect. The effect was observed from 50 μIU/mL, which is certainly attainable in the plasma of equids following a meal containing non-structural carbohydrates such as grain (Bamford et al., 2015). The maximum effect was evident at an insulin concentration of 1,000 μIU/mL and was similar at 2,000 μIU/mL; 1,000 μIU/mL was the plasma concentration achieved when insulin was administered by IV infusion to cause laminitis (Asplin et al., 2007; De Laat et al., 2010). Therefore it is quite conceivable that insulin may stimulate lamellar epithelial cells at both high physiological and supra-physiological levels, although it is unlikely that it would have any physiologically significant effect when it is within the ‘normal’ resting plasma concentration range (the normal fasting plasma concentration of insulin for horses and ponies is usually considered to be below 20 μIU/mL (Frank et al., 2010) and most laboratories quote a ‘normal’ reference range of below around 36 μIU/mL).

Fasting insulin levels are often significantly increased in ponies and horses with EMS and also in endocrinopathic laminitis cases. In ponies predisposed to laminitis and kept on pasture, mean baseline plasma insulin concentrations were greater than 30 μIU/mL and after feeding a meal containing one g/kg glucose, insulin levels to rose to a mean of 989 μIU/mL (Borer et al., 2012). In a study where mild laminitis was induced in several ponies using a dietary challenge high in non-structural carbohydrates (12 g NSC/kg BW daily split into three meals, fed for up to 18 days), those developing laminitis exhibited a mean serum insulin concentration of 385 μIU/mL 4 h after consuming each meal (Meier et al., 2018). These values are within the range capable of stimulating IGF-1 receptor activation, based on the current in vitro study. However it should be acknowledged that some individual animals may produce high insulin concentrations and not exhibit clinical signs of laminitis, so there is the distinct possibility that other factors may also be involved.

Demonstrating that blocking the IGF-1 receptors on the epithelial cells (using the blocking antibody) prevented the proliferative effect of insulin proved that it was this receptor that mediated the effect. This was further confirmed by incubating cells with recombinant human IGF-1 causing a similar proliferative response. A further experiment that could have been undertaken to further prove the absence of insulin receptors in mediating these effects would have been to examine the effects of an anti-insulin receptor antibody in this system. This represents one limitation of the current study.

In terms of IGF-1 itself being implicated in causing acute or chronic laminitis, this is probably unlikely because plasma concentrations of this hormone typically remain relatively stable throughout the day, and the main stimulus for its release from the liver is growth hormone, released from the pituitary gland, rather than dietary factors in the case of insulin. However, much of the IGF-1 in the plasma is carried bound to IGF binding proteins, and one possibility to be considered is that insulin may displace IGF-1 from one or more binding proteins, in a concentration-dependent manner. This is unlikely to be a significant factor in the present study though, as the experiments were conducted in culture medium containing just 1% adult equine serum, and of all the IGF binding proteins, insulin only appears to show affinity for IGFBP-7 (Yamanaka et al., 1997).

Recent histological studies have shown that the insulin-induced laminitis model is distinct from other forms of the condition, showing an apparent increase in basal cell mitosis as well as a lengthening of the SEL (Karikoski et al., 2014; Asplin et al., 2010). While the inflammatory form of laminitis, modelled by carbohydrate overload, is characterised by widespread separation of the lamellar epithelial cells from the basement membrane accompanied by inflammatory cell influx and matrix metalloproteinase production, the histopathology of endocrinopathic laminitis is quite distinct (Karikoski et al., 2015). A marked increase in epidermal cellular proliferation was noted on both light microscopy (Asplin et al., 2010) and electron microscopy (Nourian et al., 2009), and global separation of the lamellar dermal-epidermal interface was not apparent. Although the number of cellular hemidesmosomal attachments was reduced, the main appearance of the lesions was stretching and elongation of the SEL (Asplin et al., 2010). Therefore the dermal-epidermal junction appears to be weakened rather than becoming completely detached. Of course, secondary tissue damage leading to inflammation, combined with forces exerted by the weight of the animal, may then cause separation of the lamellae and pedal bone rotation in severe or ongoing cases.

Insulin at physiological concentrations facilitates a number of cellular responses through binding to the insulin transmembrane receptor (Wilcox, 2005). Subsequent ATP-mediated autophosphorylation of the β-subunit activates tyrosine phosphorylation of the intracellular insulin responsive substrates (IRS) (Wilcox, 2005). Tyrosine phosphorylation of the IRS then mediates the biological effects of insulin including regulation of glucose homeostasis, promoting glycogen and lipid synthesis, and inhibiting lipolysis and gluconeogenesis (Wilcox, 2005). However, due to the similarities in peptide sequence between insulin and IGF-1, insulin may also activate the IGF-1 receptor, particularly at high concentrations. The IGF-1 receptor is also a tyrosine kinase receptor, with similar α and β subunits, and leads to the activation of various intracellular signalling pathways involved in cell proliferation and activation, particularly the extracellular signal-regulated kinase pathway (ERK 1/2, also known as P42/44 MAPK) (Dupont & LeRoith, 2001). Although there are many similarities in downstream signal transduction between the insulin and IGF-1 receptors, the receptors generally tend to be present on different cell types, resulting in different responses (i.e. metabolic vs growth) (Dupont & LeRoith, 2001). Furthermore, the carboxyl terminus of the insulin receptor tends to preferentially mediate metabolic responses while the carboxyl terminus of the IGF-1 receptor particularly mediates mitogenic responses (via MAP-kinases) (Faria et al., 1994).

Previous in vitro studies in other species have shown that insulin acting via the IGF-1 receptor can stimulate the same cell signalling pathways as those produced by IGF-1 acting at the same receptor (Vigneri, Squatrito & Sciacca, 2010). In the present study, this was confirmed in equine lamellar epithelial cells using Western blotting, which demonstrated that incubation of lamellar epithelial cells with insulin caused activation of the ERK1/2 (P42/44 MAPK) cell signalling pathway, as did IGF-1. The extracellular signal-regulated kinase pathway is involved in mediating cellular responses to a diverse array of stimuli, leading to cell proliferation, differentiation and other processes involving gene expression. In order to become active, these enzymes need to become phosphorylated on both the threonine and tyrosine residues of their activation loop. Therefore enzyme activation may be assessed in cell lysates by Western blotting using antibodies specific to the phosphorylated form of the enzyme (Zhang et al., 2001). This pathway has previously been examined in epidermal lamellar tissues from the carbohydrate overload laminitis model, where it was found to be increased at the onset of Obel grade 1 signs (Gardner et al., 2015). Furthermore, lamellar tissue ERK1/2 activation, along with involvement of other signalling components, has also been observed in a dietary challenge model of laminitis and also an insulin infusion model (Lane et al., 2017).

As with the cell proliferation experiments, insulin concentrations of 1,000 and 2,000 μIU/mL both resulted in a significantly increased ratio of phospho:total ERK. Therefore further evidence is provided to support the hypothesis that activation of the IGF-1R by high concentrations of insulin can bring about the activation of epithelial cells of the epidermal lamellae, with the end result being the stimulation of mitosis. It was noted that there was a discrepancy between the lowest concentration of insulin that caused cell proliferation effects (50 μIU/mL) and the concentration required to cause measureable and statistically significant activation of the ERK signalling pathway as measured by Western blotting (1,000 μIU/mL). However, this was most likely due to the difference in sensitivity of the assays. The differences in band density between unstimulated cells and those stimulated with high concentrations of insulin in vitro are relatively slight, because in a cell culture situation there is already significant basal activation of the ERK pathway. A more sensitive method might demonstrate subtle increases in ERK activation associated with the lower range of insulin concentrations, or alternatively there may be other signalling pathways working synergistically with the ERK pathway to cause the mitotic effects.

It should be recognised that mitosis itself may not be the actual event which results in the loss of attachments between the epidermal epithelial cells and the basement membrane with subsequent weakening of the dermo-epidermal interface. There is likely to be a process of change leading ultimately to mitosis in a proportion of cells. Before that however, among the other changes and processes that are triggered within these cells may be the loss of hemidesmosomal attachments or changes to the cytoskeleton. Loss of hemidesmosomal attachments has been postulated to be an important event in the pathogenesis of laminitis (French & Pollitt, 2004). Further work is necessary to investigate the other cellular changes that are stimulated by IGF-1 receptor activation in this particular tissue, and to determine whether this causes the weakening and stretching of the SEL observed in endocrinopathic laminitis (Karikoski et al., 2014; Asplin et al., 2010).

## Conclusions

The presence of IGF-1 receptors on the basal epithelial cells of the epidermal lamellae and the fact that high but physiological concentrations of insulin can stimulate these cells via this receptor may be very significant in the pathophysiology of endocrinopathic and pasture associated laminitis. This would explain the apparent causal link between hyperinsulinaemia and laminitis and is consistent with the presence of mitotic figures in the epithelial cells examined from the tissues of horses and ponies exposed to supra-physiological intravenous infusions of insulin (Patterson-Kane, Karikoski & McGowan, 2018). Further work will determine how IGF-1 receptor-mediated cell stimulation might lead to weakening and elongation of the SEL. However, selective blockade of the IGF-1 receptor may be a potential therapeutic target for preventing this form of laminitis. Furthermore, identification of the ERK 1/2 signalling pathway also suggests further options for modulation and therapy to limit the severity and impact of endocrinopathic laminitis in the future. All of the tissues and cells used in the present study were taken from normal horses with no evidence of hyperinsulinemia or laminitis, therefore several future studies must be completed to verify and extrapolate upon these preliminary findings before the results can be applied to clinical case management.

## Supplemental Information

10.7717/peerj.5945/supp-1 Supplemental Information 1 Comparison of immunohistochemical staining for the IGF-1 receptor between chickenand rabbit polyclonal anti-IGF-1R primary antibodies, and immunohistochemical staining for the insulin receptor. Supplementary Figure 1: (A) rabbit polyclonal anti-IGF-1R primary antibody (Santa Cruz Biotechnology). (B)chicken polyclonal anti-IGF-1R primary antibody (Abcam, Cambridge, UK). (C)negative control (no primary antibody). (D) isotype-matched control antibody (chicken IgY; Abcam).
Supplementary Figure 2: IHC staining for the insulin receptor using a mouse monoclonal anti-IR primary antibody (Abcam, Cambridge, UK).

10.7717/peerj.5945/supp-2 Supplemental Information 2 Raw data supplementary file. Raw data from cell proliferation experiments (Figures 1–3) and Western blot densitometry (Figure 4). Values are expressed relative to control.

10.7717/peerj.5945/supp-3 Supplemental Information 3 Original Western blot. Western blot showing phosphorylated P42/44 ERK in cells stimulated with IGF-1 or insulin.

## Additional Information and Declarations

### Data Availability

The following information was supplied regarding data availability: The raw data is included as a Supplemental File.
