# Supplementary material for: The effect of insulin on equine lamellar basal epithelial cells mediated by the insulin-like growth factor-1 receptor
Source: PeerJ. 2018 Nov 29;6:e5945. doi: 10.7717/peerj.5945 (PMC6275117; doi:10.7717/peerj.5945)
Supplement: Supplemental Information 1 — Supplementary Figure 1: (A) rabbit polyclonal anti-IGF-1R primary antibody (Santa Cruz Biotechnology). (B)chicken polyclonal anti-IGF-1R primary antibody (Abcam, Cambridge, UK). (C)negative control (no primary antibody). (D) isotype-matched control antibody (chicken IgY; Abcam). Supplementary Figure 2: IHC staining for the insulin receptor using a mouse monoclonal anti-IR primary antibody (Abcam, Cambridge, UK). [file peerj-06-5945-s001.pdf]

Supplementary Figure 1. Comparison of IHC staining for the IGF-1 receptor between the chicken polyclonal anti-IGF-1R primary antibody used in the current paper and the rabbit polyclonal anti-IGF-1R primary antibody that was used by Burns et al (2013; ref 11) and was previously validated for IHC on paraffin-embedded tissue by Yoon et al (2011; ref 22).
Panel a): rabbit polyclonal anti-IGF-1R primary antibody (Santa Cruz Biotechnology)
Panel b): chicken polyclonal anti-IGF-1R primary antibody (Abcam, Cambridge, UK)
Panel c): negative control (no primary antibody)
Panel d): isotype-matched control antibody (chicken IgY; Abcam)

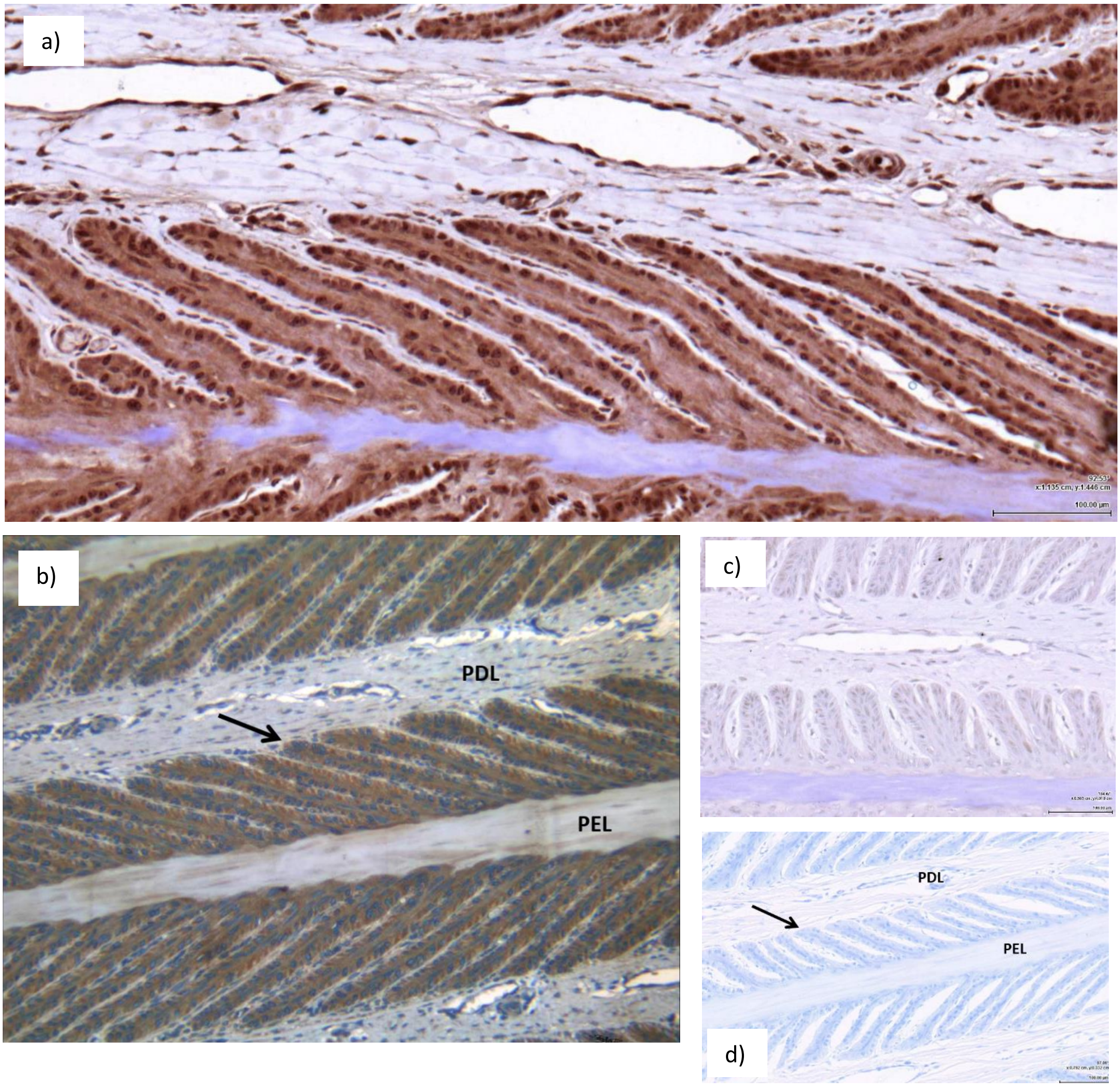

It can be seen from these images that the rabbit polyclonal antibody (Panel a) exhibits what is likely to be some non-specific staining, including all cells within the vascular dermal lamellae, as well as the cell nuclei of the basal lamellar epithelial cells. The chicken polyclonal antibody used in the present study (Panel b) mainly stains the lamellar epithelium (cytoplasm only, not nuclei), with only some minor staining of the vascular endothelium and smooth muscle within the dermal lamella. The equine insulin receptor however, is located solely on the vascular endothelium (see Supplementary Figure 2 below).

Supplementary Figure 2. IHC staining for the insulin receptor using the mouse monoclonal anti-IR primary antibody (Abcam, Cambridge, UK) previously used by Burns et al (2013; ref 11).

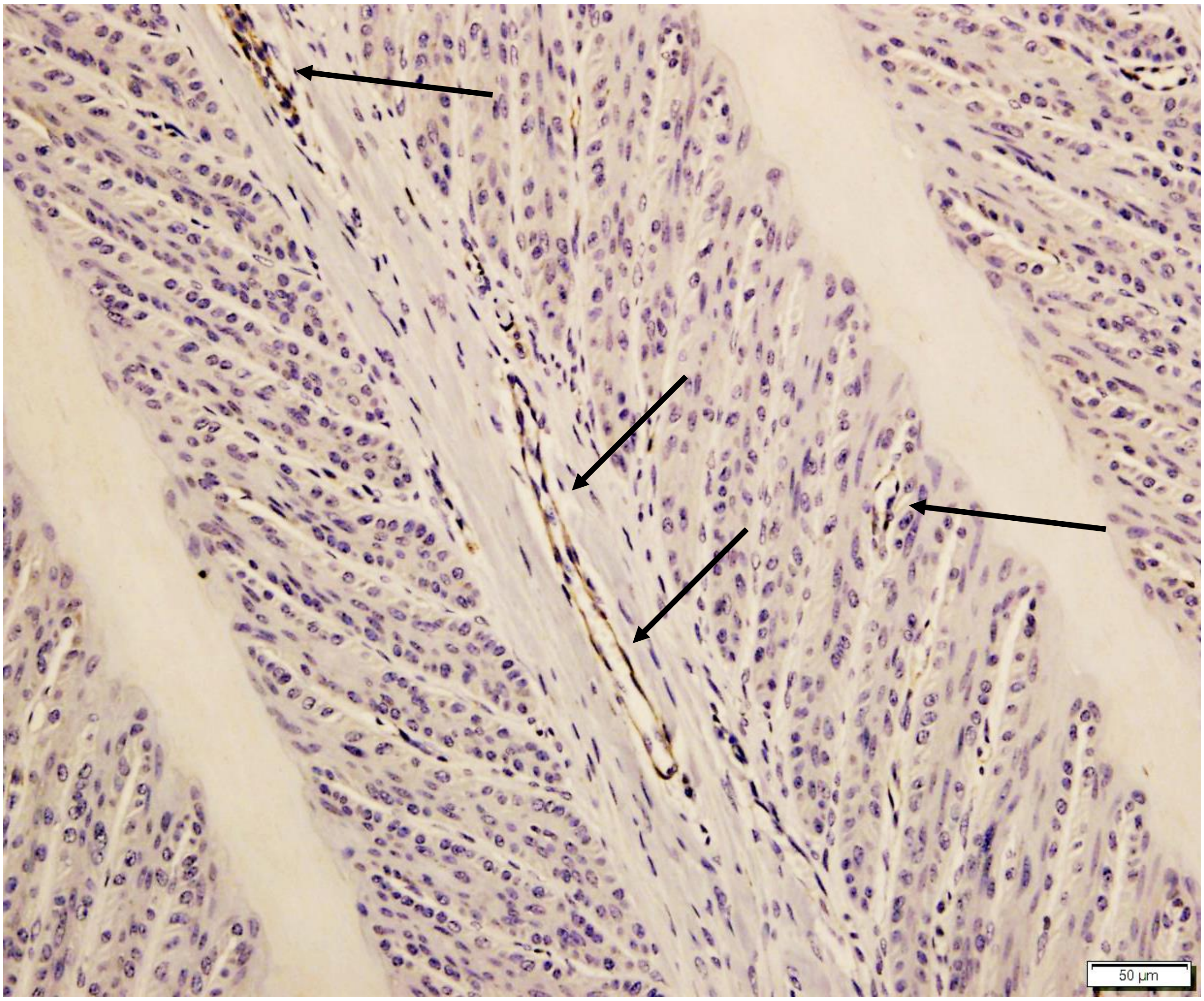

Consistent with the previous findings by Burns et al (2013; ref 11), the image shows that the insulin receptor is only found on the vascular endothelium in this tissue, and is not apparent on the epidermal lamellar epithelial cells.
