# Supplementary figures and images for: The effect of insulin on equine lamellar basal epithelial cells mediated by the insulin-like growth factor-1 receptor
Source: PeerJ. 2018 Nov 29;6:e5945. doi: 10.7717/peerj.5945 (PMC6275117; doi:10.7717/peerj.5945)

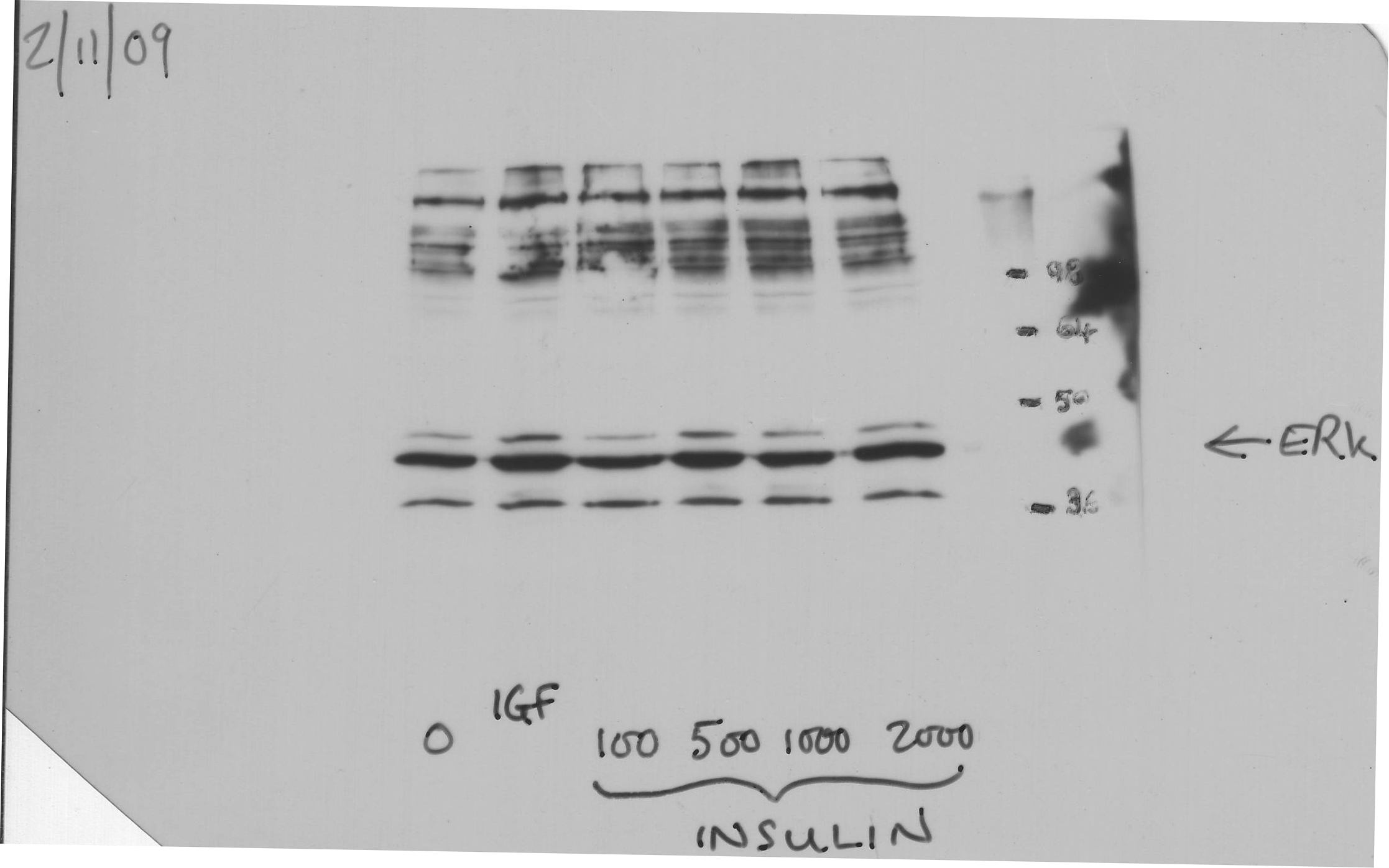

Supplement: Supplemental Information 3 — Western blot showing phosphorylated P42/44 ERK in cells stimulated with IGF-1 or insulin. [file peerj-06-5945-s003.jpg]
